# Disease Selection: The Way Disease Changed the World

**DOI:** 10.3201/eid2301.161590

**Published:** 2017-01

**Authors:** John Goldman

**Affiliations:** Pennsylvania State University College of Medicine, Hershey, Pennsylvania, USA

**Keywords:** infectious diseases, viruses, bacteria, vector-borne infections

Disease Selection: The Way Disease Changed the World by Roger Webber is written in the tradition of Rats, Lice and History by Hans Zinsser; Guns, Germs and Steel: The Fates of Human Societies by Jared M. Diamond; and Viruses, Plagues, and History by Michael B.A. Oldstone. These volumes focus on the role of diseases, particularly infectious diseases, in history. Compared with the others, however, Dr. Webber’s book is more focused on the effects of diseases on natural selection and evolution. These factors sometimes work in parallel and sometimes in opposition but always in highly complex and often unpredictable ways. The book is clearly written for a knowledgeable lay audience, a difficult task when covering evolution, microbiology, a wide variety of diseases and modes of their transmission, and immunologic responses to those diseases.

The book begins with a brief review of the origins of life on earth, including a discussion of the earliest life forms, the archaea and bacteria. Dr. Webber then describes the development of the various means of reproduction and their effects on the evolution of species and increasing complexity of life forms. In the first chapter, he covers the evolution of sexual reproduction and its effect on survival of species. Later, he discusses the role of diseases and the likely influence of endogenous viruses in the evolution of higher life forms, including humans.

Human ancestors originated in Africa and, to this day, this continent contains a unique variety of infectious agents that have likely effected human evolution. Malaria, for example, has probably led to the ability of populations to sustain sickle cell anemia because of the relative resistance of sickle red cells to *Plasmodium* spp., the cause of malaria. When the role of mosquitoes in transmission of malaria and other diseases was recognized in the 19th century, the complexity of biological relationships at all levels was revealed. For instance, because mosquitoes are damaged by blood infected with filarial parasites, those mosquitoes are less likely to transmit other disease agents such as those that cause malaria.

Smallpox, plague, influenza, and other diseases have killed large percentages of the world’s populations at various times in the past. When smallpox and measles were brought to the New World, by Europeans who were carrying variola and measles viruses, indigenous populations in South America suffered great loss of life. After these epidemics, mortality rates for survivors and their progeny were lower than for newly exposed persons who became ill. This lends support to the concept of selection of populations resistant to diseases after substantial proportions of the populations have been killed by these diseases. Later chapters deal with the effects of war, breast feeding, diet, climate change, migration, and domesticated animals on the evolution of human beings.

Dr. Webber has succeeded in the ambitious task of describing the evolution of a broad range of organisms and their interrelationships during that process, with special emphasis on how diseases have influenced the evolution of humans. The book is clearly written, and many will find the index and extensive references useful. It will be an enjoyable read for audiences with a broad range of expertise in the biological sciences.

